# Assessment of the Quality and Readability of Online Resources on Corneal Transplantation

**DOI:** 10.7759/cureus.70819

**Published:** 2024-10-04

**Authors:** Ali Mesen, Selma Meşen

**Affiliations:** 1 Ophthalmology, Kahramanmaras Sutcu Imam University, Kahramanmaraş, TUR; 2 Ophthalmology, Turkoglu Dr. Kemal Beyazıt State Hospital, Kahramanmaras, TUR

**Keywords:** cornea transplantation, keratoplasty, readability, reliability, websites

## Abstract

Purpose: The aim of this study is to evaluate the quality and readability of corneal transplant websites.

Methods: We examined internet resources related to corneal transplantation. The search terms "cornea transplantation," "keratoplasty," "keratoprothesis," and "eye transplantation" were searched on www.google.com. A total of 200 websites were scanned, with 50 websites for each search term. In the final evaluation, 113 websites were included in the study. DISCERN and the Journal of the American Medical Association (JAMA) scales were used to evaluate the websites' quality. The Flesch-Kincaid Grade Level (FKGL) and Simple Measure of Gobbledygook (SMOG) were used to evaluate the readability of the information. The presence of the Health On the Net Foundation Code of Conduct (HONCode) certification on websites was examined in terms of quality.

Results: The total DISCERN score was 42.47 ± 17.06, and the score for the JAMA was 1.58 ± 1.52. The FKGL and SMOG scores were found to be 9.19 ± 2.08 and 8.20 ± 5.23, respectively. Most of the websites we examined related to corneal transplantation were profit websites. These websites had low JAMA and DISCERN scores but relatively high readability levels. They were sites that provided low-quality information and had more financial bias and conflict of interest. Journal and book websites were found to have higher readability scores. Journal and book-sourced websites with higher FKGL and SMOG scores were more difficult to read.

Conclusion: Most of the websites exhibit financial bias, and as a result, they contain low-quality information. Online information must be more regulated for patients to easily access accurate, high-quality information.

## Introduction

Corneal diseases are a major cause of reversible blindness that can be treated with a healthy donor corneal transplant [1]. Corneal blindness is the third leading cause of blindness worldwide, following cataracts and glaucoma, and affects 10 million people with bilateral corneal blindness [2]. Penetrating keratoplasty is the full-thickness excision of the cornea and replacement with donated tissue. Due to the absence of a vascular system in the cornea, the risk of graft rejection is low, making it the most successful tissue transplant in the human body [1,3]. Corneal transplantation is the most commonly performed tissue transplant in the world. In cases where corneal damage causes severe deterioration, which affects the quality of life, corneal transplantation can restore vision [1,2]. Corneal transplantation can be performed for degenerative, dystrophic, infectious, and inflammatory corneal disorders, as well as corneal damage secondary to ocular surface disease. Recently, new lamellar transplantation surgery forms have been adopted in corneal transplantation, which only replaces the diseased layers of the cornea. Deep anterior lamellar keratoplasty replaces penetrating keratoplasty for disorders affecting the corneal stromal layers and eliminates the risk of endothelial rejection. Selective replacement of the corneal endothelium with endothelial keratoplasty has resulted in faster and more predictable visual outcomes in patients with endothelial disease [4].

With the emergence of the internet, it has been used as a tool for people to have easy access to information, including health information. Only about 7% of adults do not use the internet, and most internet users use the internet to learn more about their health conditions and potential treatments [5]. In a recent study, it was speculated that various online platforms might be instrumentalized to raise awareness about cornea donation [6]. A study showed that 35% of United States adults use the internet to gather health information about themselves or someone else with a medical condition. Of these internet users, 46% also get advice from a healthcare professional. In contrast, 38% of those who went online for health information said they had taken care of their own health at home [7]. However, with the amount of health-related websites or articles available on the internet, it has become quite complex for people to access accurate information. Studies show that the quality of health information found on the internet is highly variable [8]. The information found on the internet may be inaccurate or misleading. As a result of patients taking into account and acting on incorrect information, it may cause failure of treatment or irreversible damage to the treatment [9]. A recent study evaluating health information on the internet reported that 40% of the included websites contained incorrect information [10]. Due to the high use of the internet for health information and many people relying on the information they find online, it is important to determine whether the information provided is appropriate and reliable [11].

The capacity of people from different socioeconomic and cultural classes to acquire information also differs. Low health literacy is not an exception to its negative impact on health in ophthalmology. A recent study showed that in general, glaucoma education materials are written at the recommended 11-12th grade literacy level rather than the 7th-grade literacy level [12]. Patients can use internet resources to evaluate treatment options for diseases related to corneal transplantation. We believe it is important for patients to have access to and understand accurate information sources. In this study, we aimed to evaluate the quality and readability of information on websites related to corneal transplantation.

## Materials and methods

Data collection

For this content analysis study, the Google search engine was used on January 13, 2023. The Google search engine was chosen because it is an important interface for frequently used websites [13]. Four different search terms, "cornea transplantation," "keratoplasty," "eye transplantation," and "keratoprosthesis," were used to scan websites. A total of 200 websites were analyzed, 50 websites for each search term. To avoid being influenced by previous search results, browsing history and cookies were cleared before each search term was used. Duplicate websites, those that required membership registration or subscription, those that were not related to the topic, and those that were unreachable were excluded from the 200 websites. Each website was categorized into three categories based on the source: profit (a website with content prioritized for monetization, including government and charities), nonprofit (a website that generates support for a cause rather than revenue, including government and charity), and journals-book.

Information quality

The study used DISCERN and the Journal of the American Medical Association (JAMA) scales to assess the quality and reliability of the information. High scores from JAMA and DISCERN scoring and the presence of the HONCode seal were associated with high quality in terms of scientific content. DISCERN tool was developed in 1998 by Charnock et al. and is widely used to evaluate the content of websites [14]. It consists of 16 items that are scored from 1 to 5. The first eight items are related to general information, and the last seven items are related to treatment and treatment options. A score of 5 is given for a definite yes answer, 1 for a definite no answer, and scores of 2, 3, or 4 for partial answers. DISCERN scores can range from a minimum of 16 to a maximum of 80. The total scores can be classified as excellent (63 or above), good (51-62), fair (39-50), poor (28-38), and very poor (16-27) [15].

The JAMA scale was also developed by Silberg et al. in 1997 to evaluate the quality of a health-related website. It assesses four basic characteristics that a website should have authorship (authors, contributors, and affiliations), references (sources and citations), disclosure statements (ownership, conflicts of interest, and currency), and currency (publication dates and updates). The presence of each parameter is evaluated as 0 or 1 point, and the total score ranges from 0 to 4. High scores are associated with high content quality, and there is no categorization for scores [16]. The websites were scored by two independent reviewers trained in both DISCERN and the JAMA criteria. In case of disagreement, the authors came together and put forward their thoughts and made a final decision together.

In addition, all websites were also evaluated for Health On the Net Foundation Code of Conduct (HONCode) certification. HONCode works to identify high-quality online information, and website owners can complete the certification process by meeting the conditions that satisfy the basic principles [17].

The final three markers were used to assess the quality of online information. In this study, content that scored near a total of 80 points for DISCERN and a full 4 points for JAMA on each website was proportionally indicative of high information quality. Additionally, websites displaying the HonCODE seal were considered to have high-quality content.

Readability

Readability is the level of understanding at which a person reads a text, as evaluated by systematic formulas [15]. In addition to the quality of information in the study, a readability analysis was conducted to determine how the information could be perceived by readers. Flesch-Kincaid Grade Level (FKGL) and Simple Measure of Gobbledygook (SMOG) formulas were used for this analysis. Readability can be defined as a person's ability to understand a text. The website (https://www.webfx.com/tools/read-able/) was used for measuring readability. The readability indicators used were FKGL and SMOG. High indicators indicate low readability of the website. The formulas used to calculate readability indicators are shown in Table 1 [18].

**Table 1:** Readability formulas [14] FKGL: Flesch-Kincaid Grade Level; SMOG: Simple Measure of Gobbledygook

Parameters	Formula
FKGL	(11.8 × syllables/words ) + (0.39 × words/sentences) − 15.59
SMOG	1.0430 × (σ) x (30/sentences) + 3.1291 σ = number of words greater than or equal to 3 syllables

Elevated indicators are frequently associated with a decreased level of readability on the website. This correlation suggests that as certain evaluative metrics increase, the accessibility of the content to a broader audience diminishes, potentially hindering user comprehension and engagement.

Statistical analysis

The statistical program IBM SPSS Statistics for Windows, Version 15 (Released 2006; IBM Corp., Armonk, New York, United States) was used. The values were presented as mean, standard deviation, median, minimum, and maximum. For comparing continuous parameters that were not normally distributed in two categories, the Mann-Whitney U test was applied. For comparing categorical variables, the chi-square test was used. The interrater reliability for DISCERN and JAMA was assessed, and Cohen's kappa values were calculated.

Ethics committee

As the data used in this study were publicly available and did not involve human or animal subjects, ethical approval was not required. This study has complied with data privacy laws or guidelines. The principles of the Helsinki protocol have been applied.

## Results

**Figure 1:**
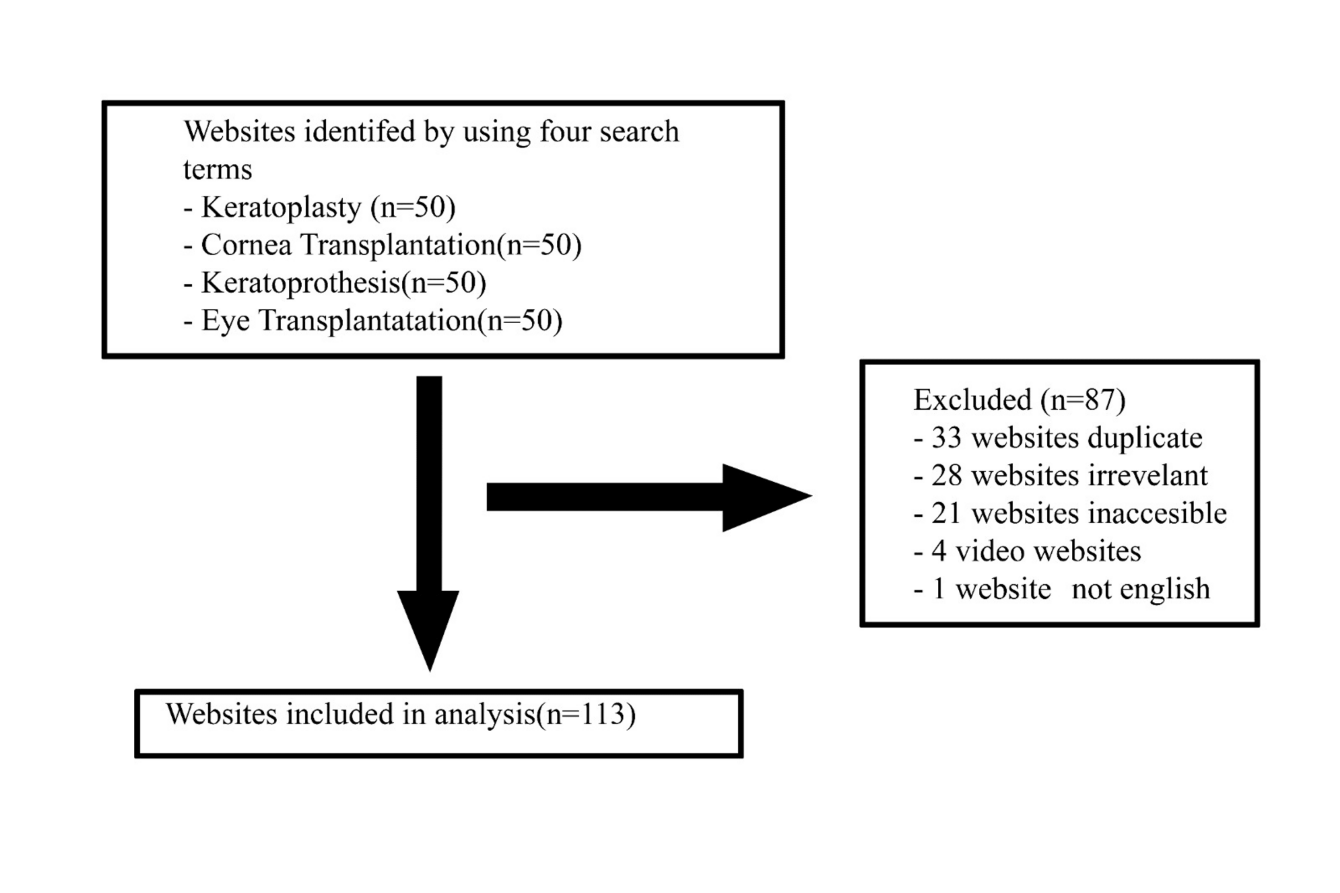
Flowchart of study

A total of 200 websites were initially included, but 28 were found to be irrelevant, 21 were inaccessible, 33 were duplicates, one was not in English, and four were video sites, which were excluded from the final analysis. As a result, a total of 113 websites were included in the final analysis (Figure 1). The inter-rater reliability kappa values were calculated as 0.78 for JAMA and 0.70 for DISCERN. The total evaluation scores were found to be 42.47 ± 17.06 for DISCERN and 1.58 ± 1.52 for JAMA. The highest scores for the subquestions of the DISCERN tool were given to "Is it relevant?" (4.27 ± 1.03) and "Does it achieve its aims?" (3.41 ± 1.16), while the lowest score was given to the question "Does it describe how the treatment choices affect the overall quality of life?" (2.06 ± 1.29) (Table 2). Of the 113 websites included in the study, 66 were for-profit, 29 were nonprofit, and 18 were part of the journal-book group. It was observed that 12.38% (n = 14) of these websites had the HONCode seal. This rate was determined to be 27.8% in the journal group, 13.8% in the nonprofit group, and 7.6% in the for-profit group (p = 0.068).

**Table 2:** DISCERN, JAMA, and readability indicators' grade level JAMA: Journal of the American Medical Association

	Mean ± SD
DISCERN	42.47 ± 17.06
1. Are the aims clear?	3.36 ± 1.16
2. Does it achieve its aims?	3.41 ± 1.16
3. Is it relevant?	4.27 ± 1.03
4. Is it clear what sources of information were used to compile the publication (other than the author or producer)?	2.40 ± 1.79
5. Is it clear when the information used or reported in the publication was produced?	2.37 ± 1.78
6. Is it balanced and unbiased?	2.73 ± 1.45
7. Does it provide details of additional sources of support and information?	2.35 ± 1.30
8. Does it refer to areas of uncertainty?	2.34 ± 1.26
9. Does it describe how each treatment works?	2.82 ± 1.30
10. Does it describe the benefits of each treatment?	2.57 ± 1.31
11. Does it describe the risks of each treatment?	2.38 ± 1.30
12. Does it describe what would happen if no treatment is used?	2.08 ± 1.29
13. Does it describe how the treatment choices affect overall quality of life?	2.06 ± 1.29
14. Is it clear that there may be more than one possible treatment choice?	2.35 ± 1.24
15. Does it provide support for shared decision-making?	2.38 ± 1.20
16. Based on the answers to all of the above questions, rate the overall quality of the publication as a source of information about treatment choices	2.62 ± 1.16
JAMA	1.58 ± 1.52
FKGL*	9.19 ± 2.08
SMOG*	8.20 ± 5.23

The readability indicators for all websites were found to be 9.19 ± 2.08 for FKGL and 8.20 ± 5.23 for SMOG. When evaluated separately for FKGL and SMOG, it was observed that the highest rate was in the journal-book group and the lowest rate was in the for-profit group (p = 0.001 and 0.002, respectively).

The DIRCERN score was found to be 67.5 for the journal-book group, 45 for the nonprofit group, and 35 for the profit group, and there was a significant difference between all groups based on subgroup analysis (p < 0.001). Similarly, the JAMA score was found to be highest in the journal-book group and lowest in the profit group (p < 0.001). When groups were compared, the readability indicators SMOG and FKGL were found to be highest in the journal-book group and lowest in the profit group. However, the high readability scores of journal-book websites indicate low readability, while the low readability scores of profit websites indicate high readability levels (Table 3).

**Table 3:** Quality and readability scores of website material according to sources n: Number; %: percentage; FKGL: Flesch-Kincaid Grade Level; SMOG: Simple Measure of Gobbledygook; HONCode: Health On the Net Foundation Code of Conduct Data are expressed as median (minimum-maximum) *Chi square

	Profit (n = 66)	Nonprofit (n = 29)	Journal-book (n = 18)	p
DISCERN	35 (16-61)	45 (16-80)	67.5 (44-80)	<0.001
JAMA	0 (0-2)	2 (0-4)	4 (3-4)	<0.001
HONCODE, n (%)	5 (7.6)	4 (13.8)	5 (27.8)	0.068*
FKGL	8.45 (6.1-17)	9 (5.8-13)	10 (8-17)	0.001
SMOG	7.20 (5.7-61)	7.50 (5.90-11.70)	8.60 (6-14)	0.002

We observed that websites with HONCode certification had higher DISCERN and JAMA scores (p-values of 0.016 and 0.388, respectively). There was no significant difference in DISCERN and JAMA scores between first-page websites and other websites (p-values of 0.513 and 0.111, respectively). There was no significant difference in SMOG and FKGL readability measures between websites with HONCode certification and first-page websites (Table 4).

**Table 4:** Comparison of websites with HONCode stamp and on the first page with some parameters FKGL: Flesch-Kincaid Grade Level; SMOG: Simple Measure of Gobbledygook; HONCode: Health On the Net Foundation Code of Conduct Data are expressed as median (minimum-maximum)

Parameter	HONCode + (n = 14)	HONCode - (n = 99)	p	First page websites (n = 32)	Other websites (n = 81)	p
DISCERN	48 (22-80)	39 (16-80)	0.016	42.5 (16-80)	40 (16-80)	0.513
JAMA	2 (0-4)	1 (0-4)	0.388	2 (0-4)	1 (0-4)	0.111
FKGL	8.55 (6.5-11)	9.00 (5.8-17)	0.368	9.00 (5.8-17)	9.00 (6.1-17)	0.572
SMOG	7.95 (5.8-61)	7.50 (5.7-14)	0.707	7.85 (5.9-61)	7.30 (5.7-14)	0.169

## Discussion

Recently, the internet has been used more frequently by both healthcare professionals and patients to access information. Due to the high use of the internet for health information and many individuals trusting the information they find online, it is important to determine whether the information presented is appropriate and reliable. The lack of standardization and regulation in current health information often leads to significant differences in the quality of published materials [19].

This study is the first to evaluate the quality and readability of information on "corneal transplantation" on the internet, as far as we have seen in the literature. This study, which examined information quality and readability at the same time, revealed that there are many for-profit sites with low-quality information. We determined that the average DISCERN score in our study was 42.47 ± 17.06. DISCERN evaluates the quality of health information provided on websites based on various factors, such as clearly listing the information sources, being balanced and unbiased, listing both the benefits and risks of each treatment option comprehensively, and supporting shared decision-making on the site. The ideal website should have scored 80 points, but none of the analyzed sites, including academic sites, were able to achieve this success. There were significant differences in DISCERN scores among corneal transplant-related websites. Particularly, websites with high scores were mostly found in journal-book websites, while those with lower scores were mostly found in profit websites. Similar studies have reported high DISCERN scores for journal-book websites, and some studies with similar results were also seen. In ophthalmology, a study based on the Google search engine calculated the mean DISCERN scores for peripheral iridotomy and trabeculectomy as 44 and 43.7, respectively. It was observed that even some websites with high DISCERN scores could contain incorrect and biased information. In the study conducted on websites in the field of calcaneal spur and plantar fasciitis, the average total DISCERN score was found to be 50.52 [13,15]. Factors such as lack of regulation within websites, the presence of misleading advertisements, and the influence of personal biases in online health content can affect information quality, security, and scores.

In our study, the average JAMA score was found to be 1.52 ± 1.58. So, on average, most websites met less than half of these four items. The JAMA score received a score of 0-2 on profit sites, while it received a score of 3-4 on journal-book sites. This leads to more questioning of the reliability of information on profit sites, which are more accessible and read by patients.

The HONcode is an initiative established to ensure that health-related websites meet certain ethical standards. HON, a Switzerland-based organization, aims to provide users with safe, accurate, and up-to-date health information on the internet. The HONcode allows websites to be labeled as "HONcode certified" if they meet certain standards, including accuracy, openness, sources of information, privacy policies, and user-friendliness. These criteria have been established to ensure that websites provide reliable and accurate information [17]. In our study, we found that only 12.38% of the websites we evaluated had the HONcode certification. When compared to other studies conducted in this area, our findings indicate a lower percentage than those reported in previous studies (respectively, 25.72% and 80%) [15,20].

Our study found that the most common websites were profit websites, with 66 out of 113 websites evaluated classified as profit websites. The low accuracy and quality of information on profit-based websites make it difficult for patients to access correct and reliable information. Studies have also shown similar results in this regard [15]. It was observed that profit websites had the lowest HONCode certification, DISCERN, and JAMA scores. Unsurprisingly, their readability levels were higher than those of journal-book and nonprofit websites. Similar results have been found in other studies when the literature is reviewed [21]. The role of healthcare professionals in guiding patients toward trusted online resources is of paramount importance. In today’s digital age, patients frequently turn to the internet for health-related information. However, the accuracy and reliability of such information are not always assured. This is where healthcare professionals play a critical role in directing their patients to reliable online sources. Such guidance helps patients better understand their health conditions and make more informed decisions. This is especially crucial for individuals with low health literacy, as they may have difficulty comprehending medical terms or treatment plans. By providing accurate information in a clear and accessible manner, healthcare professionals can bridge this gap and enhance patient education. Moreover, it is essential to highlight the role of clinicians in addressing the challenges identified in the study’s findings. These challenges often arise from communication barriers between patients and the healthcare system. Clinicians are uniquely positioned to close these gaps and improve the effectiveness of patient care processes. By encouraging the appropriate use of digital technologies and trustworthy online resources, clinicians can empower patients to engage more actively and knowledgeably in their healthcare. This guidance not only improves patients' access to reliable information but also encourages them to take a more active approach to managing their health [9,15,18].

In this study, we used the FKGL and SMOG evaluations to assess the readability of websites. The FKGL score was originally developed in 1975 to evaluate the readability of military guides for the US Navy [22]. A score of less than 8 is important for universal accessibility and readability [23]. FKGL emphasizes sentence length rather than word length, while the SMOG focuses only on multisyllable word content and analyzes the text on a lexical, rather than syntactical basis. SMOG is only valid in English and is applicable to health information studies. A suitable SMOG score for universal readability is 10 [21,23,24]. In our study, the FKGL and SMOG score values were found to be 9.19 ± 2.08 and 8.20 ± 5.23, respectively. This means that most of the evaluated websites are at the 9th-10th grade reading levels and may be more difficult for patients to understand. In a study on vocal cord paralysis, FKGL, and SMOG scores were reported as 12.96 ± 3.28 and 15.65 ± 3.57, respectively, indicating a reading level above 12th grade [25]. A study evaluating the readability of internet-based patient education materials on recent nasal septoplasty reported that the readability level was at a 10th-grade level [18].

Relatively, the for-profit websites had lower FKGL and SMOG scores, indicating higher readability for these sites. However, this does not necessarily mean that the information on these sites is reliable and up-to-date. To improve readability and comprehensibility, the American Medical Association recommends that patient education materials be written at the 7th-grade level or below [2].

There were limitations to this study. Although the readability assessment tools FKGL and SMOG are objective and repeatable for text-based websites, they do not analyze audio, visual, or video-based information. This limitation has also been described in other similar studies [26]. Secondly, another limitation was the use of only one search engine and the constantly changing nature of websites based on search engine results, as well as different results depending on location in different countries. Moreover, there are also potential limitations of the study, such as the dynamic nature of web content and the possibility of information evolution. Finally, while DISCERN and JAMA criteria are objective, the evaluators themselves may be subjective in their assessments.

## Conclusions

In conclusion, it can be said that the marker values for measuring information quality and readability used for this study on corneal transplantation are similar to many other studies on different specific topics in the literature. A significant portion of these websites show financial bias and provide low-quality information. It is difficult for a significant portion of the population to obtain health information from websites. To guide readers in their search for reliable information, seeking information from credible sources such as government health websites, recognized medical institutions, and peer-reviewed articles can lead to more accurate and consistent acquisition. There is a need to establish a mechanism to monitor and systematically manage the quality and readability of online information. Additionally, ensuring the quality and readability of health information is important for the protection of public health.
